# Association between serum total bilirubin and diabetic kidney disease in US diabetic patients

**DOI:** 10.3389/fendo.2023.1310003

**Published:** 2023-12-12

**Authors:** Jian-Min Lv, Xiu-E Shi, Qiong Ma, Nan Chen, Mi Fu, Jian-Zheng Liu, Qiao-Rong Fan

**Affiliations:** ^1^ Rehabilitation Science Institute, Shaanxi Provincial Rehabilitation Hospital, Xi’an, Shaanxi, China; ^2^ Health Department, Northwest Women’s and Children’s Hospital & Shaanxi Provincial Maternity and Child Healthcare Hospital, Xi’an, Shaanxi, China; ^3^ Department of Cardiology, Xijing Hospital, Xi’an, Shaanxi, China; ^4^ Department of Primary health care, Baoji Maternal And Child Health Hospital, Bao Ji, Shaanxi, China

**Keywords:** diabetic kidney disease, serum total bilirubin, cross-sectional study, NHANES (National Health and Nutrition Examination Survey), association

## Abstract

**Background:**

Bilirubin has been widely reported to be a protective factor against diabetic kidney disease (DKD) in Asian populations. However, few large-sample analyses have been conducted in American populations. This study aimed to investigate the association between serum total bilirubin (STB) level and DKD in a US diabetic cohort.

**Methods:**

This cross-sectional study enrolled participants from the National Health and Nutrition Examination Survey (NHANES) 2003–2018. Univariate and multivariate logistic regression analyses were performed to assess the association between STB level and DKD. Three models were conducted to control the potential confounding factors. Subgroup analysis was carried out for further validation.

**Results:**

Among the 5,355 participants, the median age [interquartile range (IQR)] was 62 [52–71] years; 2,836 (52.96%) were male, and 1,576 (29.43%) were diagnosed with DKD. In the entire cohort, no significant association between STB level and DKD was observed in any logistic regression models (*p* > 0.05). Subgroup analysis revealed that, in U.S. diabetic males, STB levels > 11.98 µmol/L were associated with a nearly 30% lower risk of DKD than STB levels ≤ 8.55 µmol/L. Additionally, a moderate STB level (8.56–11.98 μmol/L) was found associated with a nearly 25% lower risk of DKD in U.S. diabetic patients over 65 years old.

**Conclusion:**

The association of STB level with DKD may depict differences across diverse populations, among which the impact of race, sex, and age requires thorough consideration and relevant inferences should be interpreted cautiously.

## Introduction

As a serious complication of diabetes, diabetic kidney disease (DKD) is characterized by persistent albuminuria and progressive decline in glomerular filtration rate (GFR) (1–3). Ultimately, it can progress to end-stage kidney disease, in which kidney function is severely impaired and patients require dialysis or kidney transplantation to sustain life (1–3). Understanding the factors associated with DKD is crucial for managing its occurrence and enhancing the quality of life of patients (1–3).

The pathogenic mechanisms of DKD are complex, among which oxidative stress plays significant roles (4–7). Briefly, abnormal glucose metabolism leads to mitochondrial dysfunction and excessive production of free radicals, which interact with the intracellular antioxidant defense system. The resulting oxidative stress can induce lipid peroxidation, DNA damage, and protein oxidation. These issues directly lead to the destruction of the glomerular filtration membrane, injury of renal tubular cells, and renal interstitial fibrosis. Moreover, oxidative stress also induces and exacerbates inflammation, further increasing the production of oxygen-based free radicals and thus creating a vicious cycle. Overall, hyperglycemia, mitochondrial dysfunction, and inflammation play significant roles in oxidative stress in DKD. These processes interact with each other, promoting the occurrence and progression of oxidative stress, ultimately leading to renal damage and the progression of DKD (4–7).

As a metabolite of hemoglobin, bilirubin functions as an effective antioxidant, protecting cells against damage from oxidative stress through interactions with the aforementioned processes (8–10). Briefly, bilirubin mitigates cellular damage of reactive oxygen species (ROS) by binding to ROS. In addition, bilirubin regulates mitochondrial function; namely, it improves respiratory-chain function, increases ATP production, and enhances cellular energy supply (11, 12). Moreover, bilirubin reduces inflammation by inhibiting the activation of inflammatory cells and the release of inflammatory mediators, achieved through the regulation of related signal pathways (11, 13).

On another front, bilirubin level can be influenced by polymorphisms in related genes, including but not limited to *UGT1A1*, *OATP2*, *HMOX1*, *BLVRA*, and *NOS3* (14–17). Among these genetic variations, some may also affect susceptibility to DKD and thus contribute to protective mechanisms. For instance, bilirubin has been shown to be related to the generation and function of endothelial nitric oxide synthase (eNOS), which is involved in the regulation of renal and glomerular hemodynamics (18). It has been reported that *NOS3*, the gene encoding eNOS, harbors genetic susceptibility to increased bilirubin levels (17). Additionally, *NOS3* polymorphisms are associated with altered susceptibility to DKD, and thus *NOS3* is a candidate target gene in the treatment of DKD (19).

In recent decades, numerous studies have reported the correlation of serum total bilirubin (STB) levels with DKD (20, 21). Although a high STB level is currently considered a protective factor against DKD, several studies have presented conflicting conclusions (20–24). Moreover, previous research has predominantly focused on Asian populations, such as Chinese, Japanese, and Korean, and data regarding European and American populations are limited (20, 21). In the studies involving Western populations by Targher G et al. (22, 23), STB was found to be either uncorrelated or positively associated with DKD risk in diabetic patients, inconsistent with the conclusions of most Asian population studies. The potential reasons behind these inconsistent results remain unclear and might be related to differences among different populations, such as variations in genetic and lifestyle factors.

Therefore, we conducted a large-sample study to investigate the association of STB level with DKD in the American population, using data from the National Health and Nutrition Examination Survey (NHANES).

## Materials and methods

### Data source and study population

NHANES is a national survey of the civilian non-institutionalized population conducted by the National Center for Health Statistics (NCHS), employing a cross-sectional, multistage, stratified, subgroup probability sampling study design on a two-year cycle. Further details on NHANES are available at https://www.cdc.gov/nchs/nhanes/. The NHANES study was approved by the NCHS Ethics Review Board in the US.

**Figure 1:**
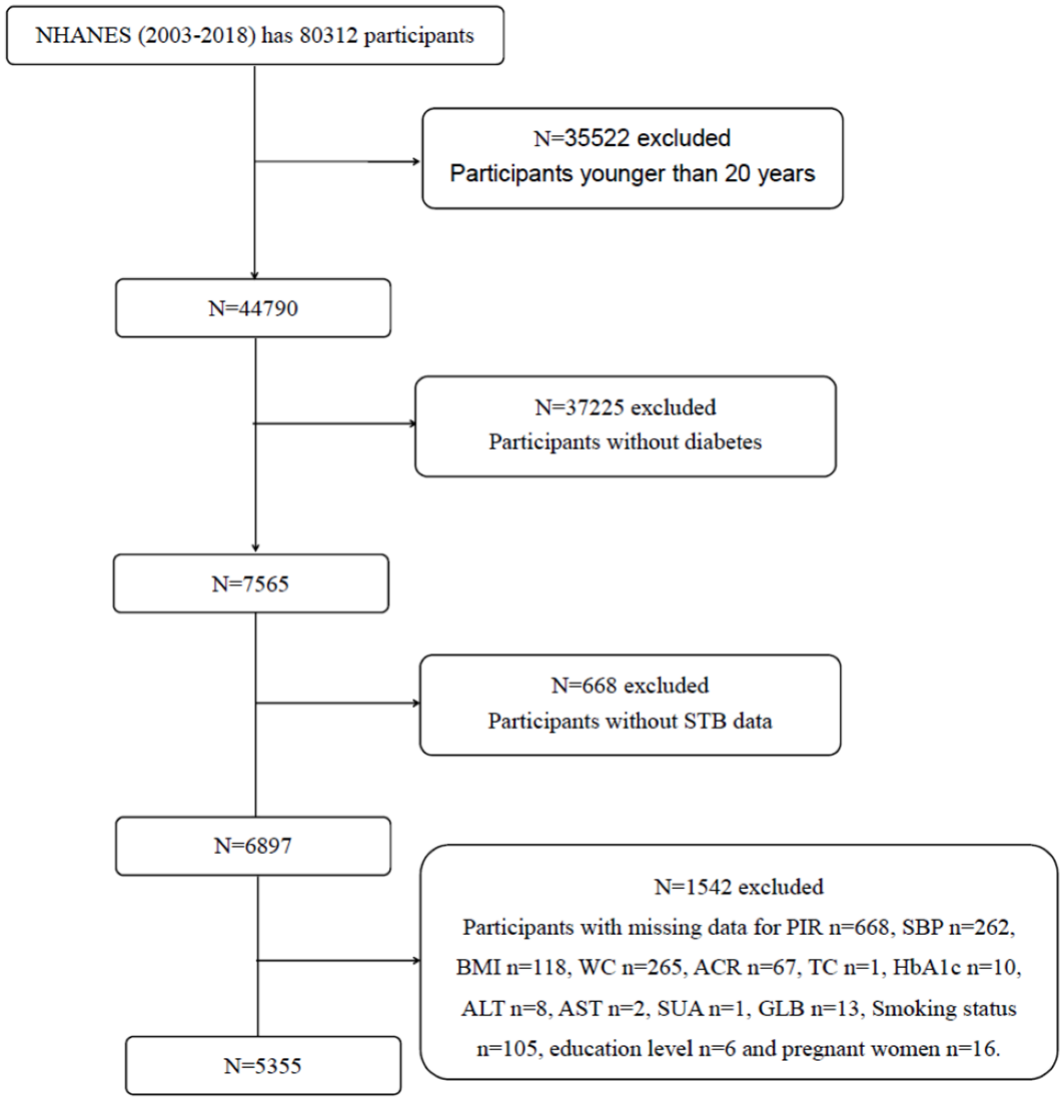
Flowchart of participant selection.

This study involved a total of 80312 participants from eight NHANES cycles spanning from 2003 to 2018. The flowchart of participant selection is provided in **Figure 1**. Eventually, 5355 patients with diabetes were enrolled. The demographics, examination results, questionnaire data, and laboratory data of the participants were extracted.

### Definition of diabetes and DKD

In accordance with the standards of the American Diabetes Association and previous research, the diagnosis of diabetes was established through (1) a previous diagnosis by medical professionals, (2) fasting plasma glucose level ≥ 7.0 mmol/L, (3) glycohemoglobin (HbA1c) value ≥ 6.5%, or (4) current treatment for diabetes (25). Participants with an albumin/creatinine ratio (ACR) ≥ 30 μg/mg were classified as DKD patients and those with an ACR < 30 μg/mg as non-DKD patients (1–3).

### Assessment of STB

As the dependent variable of this study, STB level (μmol/L) was measured using the method based on the NHANES Laboratory Procedures. Briefly, in the presence of a solubilizing agent, STB is coupled with 3,5-dichlorophenyl diazonium in a strongly acidic medium, and the intensity of the red azo dye formed is directly proportional to the STB level and can be determined photometrically (546 nm). In the present study, STB level was categorized into Q1 (≤ 8.55 μmol/L), Q2 (8.56–11.98 μmol/L), and Q3 (≥ 11.98 μmol/L) according to tertiles.

### Covariates

In accordance with previous studies, the following potential covariates were considered: age, sex, race/ethnicity, education level, poverty income ratio (PIR), body mass index (BMI), waist circumference (WC), presence of hypertension, smoking status, blood urea nitrogen (BUN) level, serum uric acid (SUA) and serum creatinine (SCR) levels, and blood levels of total cholesterol (TC), high-density lipoprotein (HDL) cholesterol, glycohemoglobin (HbA1c), albumin (ALB), alamine aminotransferase (ALT), aspartate aminotransferase (AST), gamma-glutamyltransferase (GGT), and globulin (GLB).

Among these covariates, race/ethnicity was categorized into the following five groups: Mexican American, other Hispanic, non-Hispanic white, non-Hispanic black, and other race-including multi-racial. Education level was classified as below high school, high school graduate/GED or equivalent, and college or above. BMI was categorized into normal (< 25.0 kg/m^2^), overweight (25.0 to < 30.0 kg/m^2^), and obese (≥ 30.0 kg/m^2^). Additionally, WC values > 88 cm and > 102 cm for women and men, respectively, were used to define abdominal obesity. Hypertension was defined as systolic blood pressure (SBP) ≥ 130 mmHg and/or diastolic blood pressure (DBP) ≥ 80 mmHg after repeated examination by a physician. For smoking status, participants were divided into never-smokers (individuals who never smoked or smoked < 100 cigarettes in life) and smokers (having smoked ≥ 100 cigarettes in life).

### Statistical analysis

To obtain nationally representative results, appropriate sample weights were applied to account for oversampling and nonresponse. Weighted analyses were performed following the guidelines of the NHANES.

Categorical variables were expressed as numbers (percentages). Continuous variables with a normal distribution were presented as mean ± standard deviation (SD), whereas those with an abnormal distribution were presented as median [Q1, Q3]. Weighted chi-squared tests or Kruskal-Wallis tests were employed to compare basic characteristics among the three STB groups, and weighted chi-squared tests or Mann-Whitney U tests were employed to compare basic characteristics between DKD and Non-DKD groups.

Weighted univariate logistic regression model (model 1) and multivariable logistic regression models (models 2 and 3) were employed to calculate the odds ratio (OR) and 95% confidence interval (95% CI) for the association between STB level and DKD. Model 2 was adjusted for age, gender, race, education level, and PIR. Model 3 was adjusted for the factors included in model 2 and for BMI, WC, presence of hypertension, TC, HDL, HbA1c, ALB, ALT, AST, BUN, GGT, SUA, SCR, GLB, and smoking status.

To further determine the potential effect moderators and explore the association between STB level and DKD, subgroup analysis was performed according to age, sex, race/ethnicity, education level, PIR, BMI, WC, smoking status, and presence of hypertension. Three logistic regression models were constructed.

Statistical analysis was performed using R statistical software version 4.2 (R Project for Statistical Computing). A two-sided *p* < 0.05 was considered statistically significant.

## Results

### Baseline characteristics

Among the 5355 participants included from the 2003–2018 NHANES, the median age [IQR] was 62 [52–71] years, 2836 (52.96%) were male, and 1576 (29.43%) had DKD.

**Table 1** shows the baseline characteristics based on STB levels, divided into Q1, Q2, and Q3 according to tertiles. Age, sex, race/ethnicity, PIR, BMI, WC, and levels of DBP, TC, HbA1c, HSA, ALT, AST, GGT, UA, SCR, and GLB exhibited significant differences among the various STB groups (*p* < 0.05). Participants in the Q3 group tended to be male and have a BMI < 30 kg/m^2^. Additionally, they tended to be older, with higher PIR, and higher levels of HSA, ALT, AST, GGT, UA, and SCR.

**Table 1:** Basic characteristics of the participants based on STB tertiles.

Characteristics	Total (N=5355)	Q1 (≤8.55 μmol/L) N= 2249	Q2 (8.56-11.98 μmol/L) N= 1624	Q3 (≥11.98 μmol/L) N=1482	Weighted p value
**Age, years (median [IQR])**	62 [52, 71]	61 [51, 69]	63 [53, 71]	63 [53, 73]	<0.001*
**Age ≥ 65 years, n (%)**	2280 (42.58)	845 (37.57)	746 (45.94)	689 (46.49)	0.003*
**Sex, n (%)**					<0.001*
Male	2836 (52.96)	952 (42.33)	835 (51.42)	1049 (70.78)	
Female	2519 (47.04)	1297 (57.67)	789 (48.58)	433 (29.22)	
**Race/Ethnicity, n (%)**					<0.001*
Mexican American	1033 (19.29)	384 (17.07)	344 (21.18)	305 (20.58)	
Other Hispanic	527 (9.84)	247 (10.98)	153 (9.42)	127 (8.57)	
Non-Hispanic White	1953 (36.47)	700 (31.12)	610 (37.56)	643 (43.39)	
Non-Hispanic Black	1312 (24.50)	664 (29.52)	362 (22.29)	286 (19.30)	
Other Race-Including Multi-Racial	530 (9.90)	254 (11.29)	155 (9.54)	121 (8.16)	
**Education, n (%)**					0.086
Below high school	938 (17.52)	375 (16.67)	307 (18.90)	256 (17.27)	
High school Grad/GED or Equivalent	873 (16.30)	357 (15.87)	283 (17.43)	233 (15.72)	
College or above	3544 (66.18)	1517 (67.45)	1034 (63.67)	993 (67.00)	
**PIR (median [IQR])**	1.84 [1.04, 3.48]	1.77 [0.99, 3.25]	1.81 [1.03, 3.41]	2.00 [1.12, 3.90]	<0.001*
**BMI (kg/m^2^), (median [IQR])**	31.10 [27.20, 36.00]	31.80 [27.70, 37.20]	30.80 [27.28, 35.52]	30.14 [26.67, 34.70]	<0.001*
**BMI, n (%)**					<0.001*
< 25 kg/m^2^	710 (13.26)	275 (12.23)	213 (13.12)	222 (14.98)	
25~30kg/m^2^	1566 (29.24)	572 (25.43)	496 (30.54)	498 (33.60)	
≥30 kg/m^2^	3079 (57.50)	1402 (62.34)	915 (56.34)	762 (51.42)	
**WC (cm), (median [IQR])**	107.50 [98.00, 118.60]	108.50 [98.90, 120.00]	106.90 [97.70, 117.53]	107.00 [97.20, 117.50]	0.001*
**Abdominal obesity**	4192 (78.26)	1843 (81.95)	1279 (78.76)	1070 (72.22)	<0.001*
**SBP (mmHg), (median [IQR])**	129 [118, 142]	129 [118, 143]	129 [118, 142]	128 [117, 141]	0.069
**DBP (mmHg), (median [IQR])**	70 [62, 78]	70 [62, 78]	69 [60, 77]	71 [62, 78]	0.007*
**Hypertension, n (%)**	2878 (53.74)	1224 (54.42)	882 (54.31)	772 (52.09)	0.154
**Smoker, n (%)**	2695 (50.33)	1129 (50.20)	816 (50.25)	750 (50.61)	0.141
**TC (mmol/L), (median [IQR])**	4.71 [3.98, 5.53]	4.73 [4.01, 5.51]	4.76 [4.03, 5.59]	4.65 [3.93, 5.56]	0.019*
**HDL (mmol/L), (median [IQR])**	1.19 [1.01, 1.45]	1.19 [0.98, 1.45]	1.19 [1.01, 1.45]	1.19 [1.01, 1.42]	0.103
**HbA1c (%), (median [IQR])**	6.80 [6.20, 7.90]	6.90 [6.30, 8.10]	6.80 [6.20, 7.80]	6.70 [6.10, 7.90]	0.004*
**ALB (g/L), (median [IQR])**	41 [39, 43]	41 [39, 43]	41 [39, 44]	42 [40, 44]	<0.001*
**ALT (U/L), (median [IQR])**	22 [17, 30]	20 [16, 28]	22 [17, 30]	24 [18, 34]	<0.001*
**AST (U/L), (median [IQR])**	23 [19, 28]	21 [18, 27]	23 [19, 28]	24 [21, 31]	<0.001*
**BUN (mmol/L), (median [IQR])**	5.00 [3.93, 6.78]	5.00 [3.93, 6.78]	5.00 [3.93, 6.78]	5.00 [3.93, 6.78]	0.499
**GGT (U/L), (median [IQR])**	25 [17, 39]	24 [17, 37]	25 [17, 37]	27 [18, 43]	<0.001*
**SUA (umol/L), (median [IQR])**	333.10 [279.60, 398.50]	327.10 [267.70, 386.60]	333.10 [279.60, 398.50]	345.00 [291.50, 410.40]	<0.001*
**SCR (umol/L), (median [IQR])**	79.56 [64.53, 97.24]	76.02 [61.88, 93.70]	79.56 [64.53, 98.12]	81.33 [68.95, 98.12]	<0.001*
**GLB (g/L), (median [IQR])**	30 [27, 33]	30 [27, 34]	30 [27, 33]	30 [27, 33]	<0.001*

PIR, Poverty income ratio; BMI, Body mass index; WC, Waist circumference; SBP, Systolic Blood Pressure; SDP, Diastolic blood pressure; TC, Total cholesterol; HDL, High-density lipoprotein cholesterol; HbA1c, Glycohemoglobin; ALB, Albumin; ALT, Alamine aminotransferase; AST, Aspartate aminotransferase; BUN, Blood urea nitrogen; GGT, Gamma-glutamyltransferase; SUA, Serum uric acid; SCR, Serum creatinine; GLB, Globulin. *, *p* <0.05.

**Table 2** shows the baseline characteristics based on the DKD status. Overall, age, race/ethnicity, education level, PIR, presence of hypertension, and levels of HDL, HbA1c, HSA, ALT, BUN, GGT, UA, SCR, and GLB exhibited significant differences between the DKD and non-DKD participants (*p* < 0.05). Compared with the non-DKD participants, those with DKD tended to be older, male, smoker, have an education level below high school, a BMI < 25 kg/m^2^, hypertension, and higher levels of HbA1c, BUN, GGT, UA, SCR, and GLB. The proportion of participants with DKD varied by race/ethnicity. In contrast, PIR, and levels of HDL, HSA, and ALT were higher in the non-DKD group than in the DKD group.

**Table 2:** Basic characteristics of the participants based on DKD.

Characteristics	Total (N=5355)	Non-DKD (N=3779)	DKD (N=1576)	Weighted p value
**Age (median [IQR])**	62 [52, 71]	61 [51, 69]	65 [54, 74]	<0.001*
**Age ≥ 65 (%)**	2280 (42.58)	1483 (39.24)	797 (50.57)	<0.001*
**Sex**				0.057
Male	2836 (52.96)	1931 (51.10)	905 (57.42)	
Female	2519 (47.04)	1848 (48.90)	671 (42.58)	
**Race/Ethnicity, (%)**				<0.001*
Mexican American	1033 (19.29)	695 (18.39)	338 (21.45)	
Other Hispanic	527 (9.84)	380 (10.06)	147 (9.33)	
Non-Hispanic White	1953 (36.47)	1399 (37.02)	554 (35.15)	
Non-Hispanic Black	1312 (24.50)	929 (24.58)	383 (24.30)	
Other Race-Including Multi-Racial	530 (9.90)	376 (9.95)	154 (9.77)	
**Education (%)**				<0.001*
Below high school	938 (17.52)	614 (16.25)	324 (20.56)	
High school Grad/GED or Equivalent	873 (16.30)	581 (15.37)	292 (18.53)	
College or above	3544 (66.18)	2584 (68.38)	960 (60.91)	
**PIR (median [IQR])**	1.84 [1.04, 3.48]	1.98 [1.08, 3.71]	1.60 [0.96, 2.92]	<0.001*
**BMI (median [IQR])**	31.10 [27.20, 36.00]	31.19 [27.30, 36.07]	30.90 [26.80, 35.90]	0.792
**BMI (%)**				0.087
< 25 kg/m^2^	710 (13.26)	464 (12.28)	246 (15.61)	
25~30kg/m^2^	1566 (29.24)	1127 (29.67)	439 (27.86)	
≥30 kg/m^2^	3079 (57.50)	2188 (57.90)	891 (56.54)	
**WC (median [IQR])**	107.50 [98.00, 118.60]	107.30 [98.00, 118.20]	108.20 [98.07, 119.62]	0.085
**Abdominal obesity**	4192 (78.28)	2976 (78.8)	1216 (77.2)	0.367
**SBP (median [IQR])**	129 [118, 142]	126 [116, 138]	136 [123, 153]	<0.001*
**DBP (median [IQR])**	70 [62, 78]	70 [62, 77]	70 [60, 79]	0.028*
**Hypertension (%)**	2878 (53.74)	1813 (47.98)	1065 (67.58)	<0.001*
**Smoker (%)**	2695 (50.33)	1848 (48.90)	847 (53.74)	0.006*
**TC (median [IQR])**	4.71 [3.98, 5.53]	4.73 [4.01, 5.51]	4.68 [3.96, 5.61]	0.288
**HDL (median [IQR])**	1.19 [1.01, 1.45]	1.19 [1.01, 1.45]	1.14 [0.98, 1.42]	0.023*
**HbA1c (median [IQR])**	6.80 [6.20, 7.90]	6.70 [6.10, 7.60]	7.20 [6.40, 8.70]	<0.001*
**ALB (median [IQR])**	41 [39, 43]	42 [39, 44]	41 [38, 43]	<0.001*
**ALT (median [IQR])**	22 [17, 30]	22 [17, 30]	21 [16, 29]	0.09
**AST (median [IQR])**	23 [19, 28]	23 [19, 28]	23 [19, 29]	0.404
**BUN (median [IQR])**	5.00 [3.93, 6.78]	5.00 [3.93, 6.43]	5.71 [4.28, 7.85]	<0.001*
**STB (median [IQR])**	10.26 [6.84, 13.68]	10.26 [6.84, 13.68]	10.26 [8.55, 13.68]	0.952
**GGT (median [IQR])**	25 [17, 39]	24 [17, 38]	26 [18, 42]	<0.001*
**SUA (median [IQR])**	333.10 [279.60, 398.50]	327.10 [273.60, 392.60]	345.00 [285.50, 416.40]	<0.001*
**SCR (median [IQR])**	79.56 [64.53, 97.24]	76.02 [63.65, 91.05]	84.86 [68.95, 114.92]	<0.001*
**GLB (median [IQR])**	30 [27, 33]	30 [27, 33]	31 [28, 35]	<0.001*

PIR, Poverty income ratio; BMI, Body mass index; WC, Waist circumference; SBP, Systolic Blood Pressure; DBP, Diastolic blood pressure; TC, Total cholesterol; HDL, High-density lipoprotein cholesterol; HbA1c, Glycohemoglobin; ALB, Albumin; ALT, Alamine aminotransferase; AST, Aspartate aminotransferase; BUN, Blood urea nitrogen; STB, Serum total bilirubin; GGT, Gamma-glutamyltransferase; SUA, Serum uric acid; SCR, Serum creatinine; GLB, Globulin. *, *p* <0.05.

### Association between STB level and DKD

Three logical regression models were established to analyze the relationship between STB level and DKD, and the effect value was expressed as OR and 95%CI (**Table 3**). However, no significant association between STB level and DKD was observed in any of the models.

**Table 3:** Association between STB and DKD.

Serum Total bilirubin	OR (95%CI)	p value
**Model 1**		
Q1	Ref	
Q2	1.00 (0.83,1.21)	0.996
Q3	0.92 (0.76,1.11)	0.375
**Model 2**		
Q1	Ref	
Q2	0.94 (0.77,1.15)	0.557
Q3	0.87 (0.70,1.08)	0.201
**Model 3**		
Q1	Ref	
Q2	0.94 (0.76,1.16)	0.567
Q3	0.95 (0.75,1.21)	0.691

Model 1: unadjusted. Model 2: adjusted for age, gender, race/ethnicity, education level, and PIR. Model 3: Model 2+BMI, WC, Hypertension, TC, HDL, HbA1c, ALB, ALT, AST, BUN, GGT, SUA, SCR, GLB, Smoking status.

For the validation of the outcomes, subgroup analysis was performed (**Tables 4** and **S1**). When age was stratified at 65 years old, all models indicated that for participants over 65 years old, an STB level of Q2 (8.56–11.98 μmol/L) was associated with a lower risk of DKD than Q1 (< 8.55 μmol/L). The results (OR [95%CI]) were as follows: 0.72 (0.54–0.97) for Model 1, 0.71 (0.53–0.93) for Model 2, and 0.67 (0.50–0.89) for Model 3. However, no difference was found for participants below 65 years old.

**Table 4:** Association between STB and DKD according to age and sex.

Subgroup	Q2 (8.56-11.98 μmol/L)							Q3 (≥11.98 μmol/L)						
	p for interaction	Model 1		Model 2		Model 3		p for interaction	Model 1		Model 2		Model 3	
		OR (95%CI)	p	OR (95%CI)	p	OR (95%CI)	p		OR (95%CI)	p	OR (95%CI)	p	OR (95%CI)	p
**Age (years)**	0.020*							0.872						
< 65		1.19 (0.90, 1.57)	0.221	1.14 (0.85, 1.52)	0.373	1.11 (0.83, 1.47)	0.480		0.86 (0.64, 1.16)	0.324	0.79 (0.58, 1.09)	0.151	0.79 (0.55, 1.12)	0.182
≥ 65		0.72 (0.54, 0.97)	0.028*	0.71 (0.53, 0.93)	0.016*	0.67 (0.50, 0.89)	0.007*		0.88 (0.65, 1.18)	0.381	0.88 (0.65, 1.18)	0.381	0.85 (0.62, 1.15)	0.286
**Sex**	0.971							0.095						
Male		0.97 (0.71, 1.33)	0.862	0.96 (0.71, 1.32)	0.817	0.93 (0.68, 1.28)	0.667		0.75 (0.58, 0.98)	0.032*	0.77 (0.59, 1.00)	0.049*	0.75 (0.57, 1.00)	0.047*
Female		0.96 (0.72, 1.30)	0.809	0.89 (0.65, 1.22)	0.471	0.90 (0.66, 1.25)	0.534		1.10 (0.77, 1.59)	0.592	1.03 (0.71, 1.50)	0.858	1.00 (0.68, 1.46)	0.987

*, *p* <0.05.

Furthermore, subgroup analysis revealed that for males, but not females, participants with higher STB levels (Q3, ≥ 11.98 μmol/L) had a significantly lower risk of DKD. This association was significant in Model 1 (0.75 [0.58–0.98]), Model 2 (0.77 [0.59–1.00]), and Model 3 (0.75 [0.57–1.00]). No significant association was found after stratification based on race/ethnicity, education level, PIR, BMI, WC, smoking status, and presence of hypertension (**Table S1**).

## Discussion

In this study, we investigated the association between STB level and DKD in a US diabetic cohort from the NHANES 2003–2018. Contrary to the reports on Asian populations, we observed no significant association between STB level and the risk of DKD in the entire diabetic cohort. However, subgroup analyses revealed that compared with low STB levels, high STB levels are associated with a reduced risk of DKD in US diabetic males, whereas moderate levels are associated with a reduced DKD risk in diabetics over 65 years old.

In the past twenty years, a series of cross-sectional studies have consistently suggested an association between high STB levels and a reduced risk of DKD in patients with diabetes (26–35). Concurrently, animal studies have been conducted to explore the mechanism whereby STB confers protection against DKD (36, 37). However, there have been a few studies with conflicting results, suggesting no relationship between STB level and DKD (22, 24), or even positing STB as a risk factor for DKD (23).

In a cross-sectional study using data from the NHANES 2001–2006, Targher G et al. (22) found that STB level is not correlated with GFR or albuminuria in patients with diabetes (n = 1253). As an updated study, here we used samples from NHANES 2003–2018, and our sample size was larger than that of the study by Targher G et al. (n = 5355). Our analyses on this larger and updated sample set reproduced the findings of Targher G et al., thereby reinforcing the reliability of their conclusion. Another retrospective analysis by Targher G. et al. (23) indicated a negative correlation between STB and GFR levels in an unselected outpatient cohort of Caucasian non-diabetic and diabetic adults. This finding contrasts with conclusions drawn from the majority of previous studies based on Asian populations.

Considering our findings alongside the aforementioned research results, we propose that the association of STB level with DKD may exhibit distinctions among diverse populations. We speculate that the contrasting findings between Asian and Western populations can be attributed to several factors. Firstly, genetic variations may contribute to differences in both bilirubin metabolism and DKD susceptibility. For example, the *NOS3* gene is implicated in the genetic susceptibility to elevated STB levels (17), and polymorphisms in this gene have been associated with altered DKD susceptibility (19). Although not conclusively confirmed, it cannot be ruled out that these genetic variations may influence the association between STB level and DKD. Secondly, lifestyle factors might also contribute to the observed differences. Western populations often have distinct dietary patterns, physical activity levels, and environmental exposures compared to Asian populations. These lifestyle factors may interact with STB levels and influence the development of DKD. Lastly, other factors that vary between Asian and American populations, such as comorbidities and socioeconomic status, may also confound the relationship between STB level and DKD. Further studies are needed to clarify the mechanisms and provide insights into this disparity.

In our further analysis, we found that the association between STB level and DKD varied with age and sex. Specifically, for US diabetic individuals over 65 years old, having STB levels in Q2 (8.56–11.98 μmol/L) was associated with a nearly 30% lower risk of developing DKD than those with STB levels < 8.55 μmol/L. However, this association became statistically insignificant for individuals aged > 65 years with STB levels > 11.98 μmol/L, as well as for those aged < 65 years old, regardless of STB levels. Additionally, we found that males and females differed in the correlation between STB levels and DKD. Notably, a higher STB level (> 11.98 μmol/L) was observed to be associated with a nearly 25% reduced risk of DKD in males, whereas no significant correlation was found in females. Importantly, such sex- and age-related differences in both STB level and DKD prevalence have been widely reported (38–40), as also reflected in our results (**Tables 1** and **2**).

Overall, we recommend thorough consideration of the impact of race, sex, and age when exploring the correlation between STB level and DKD, and caution in interpreting relevant conclusions. Notably, this study represents the first large-scale investigation into the relationship between STB level and DKD in the US population. The major strength of our study lies in the substantial sample size, as all the eligible participants from eight NHANES cycles (2003–2018) were included, ensuring the reliability of our conclusions.

However, our study has some limitations. Firstly, despite revealing the association between STB level and DKD in specific subgroups, establishing causation is not feasible due to the cross-sectional nature of the study. Prospective studies are necessary in the future to elucidate the causal relationship between STB level and DKD risk. Secondly, although we controlled for certain confounders in the subgroup analysis, there may still be unaccounted-for confounders not included or recorded in the NHANES. Thirdly, our study could not explore potential hereditary factors that might influence the association between STB level and DKD. Bilirubin levels exhibit significant genetic predispositions, such as hereditary hyperbilirubinemias. Additionally, hereditary susceptibility plays a role in the pathogenesis of DKD (41). Therefore, hereditary factors might influence the association between STB level and DKD. However, it is not possible to assess the family relationships among the participants and obtain information on hereditary factors in the data from the NHANES because it is a randomly sampled cross-sectional survey. We anticipate that prospective studies in the future will provide further elucidation and confirmation of the relationship between STB level and DKD.

## Conclusion

The correlation of STB level with DKD could exhibit distinctions across diverse populations, among which the impact of race, sex, and age demands thorough consideration. Compared with STB levels < 8.55 μmol/L, STB levels > 11.98 μmol/L are associated with a nearly 30% reduced risk of DKD in US diabetic males. Furthermore, a moderate STB level (8.56–11.98 μmol/L) is linked to a nearly 25% reduced risk of DKD in U.S. diabetic patients aged > 65 years. Further validation through large-scale prospective cohorts is warranted in the future.

## Data availability statement

Publicly available datasets were analyzed in this study. This data can be found here: https://www.cdc.gov/nchs/nhanes/.

## Ethics statement

The studies involving humans were approved by The National Centre for Health Statistics Ethics Review Board in the US. The studies were conducted in accordance with the local legislation and institutional requirements. The participants provided their written informed consent to participate in this study.

## Author contributions

J-ML: Data curation, Formal Analysis, Funding acquisition, Methodology, Writing – original draft, Writing – review & editing. X-ES: Project administration, Supervision, Writing – review & editing. QM: Data curation, Formal Analysis, Methodology, Writing – review & editing. NC: Data curation, Formal Analysis, Methodology, Writing – review & editing. MF: Data curation, Formal Analysis, Methodology, Writing – review & editing. J-ZL: Project administration, Supervision, Writing – review & editing. Q-RF: Project administration, Supervision, Writing – review & editing.
