# Supplementary material for: Association between serum total bilirubin and diabetic kidney disease in US diabetic patients
Source: Front Endocrinol (Lausanne). 2023 Dec 12;14:1310003. doi: 10.3389/fendo.2023.1310003 (PMC10752268; doi:10.3389/fendo.2023.1310003)
Supplement: Supplementary file 1 [file Table_1.doc]

**Table S1. Association between STB and DKD according to several general characteristics.**

| **Subgroup** | **Q2 (8.56-11.98 μmol/L)** | | | | | | |  | **Q3 (≥11.98 μmol/L)** | | | | | | |
| --- | --- | --- | --- | --- | --- | --- | --- | --- | --- | --- | --- | --- | --- | --- | --- |
|  | ***p* for interaction** | **Model 1** | | **Model 2** | | **Model 3** | |  | ***p* for interaction** | **Model 1** | | **Model 2** | | **Model 3** | |
|  |  | **OR (95%CI)** | ***p*** | **OR (95%CI)** | ***p*** | **OR (95%CI)** | ***p*** |  |  | **OR (95%CI)** | ***p*** | **OR (95%CI)** | ***p*** | **OR (95%CI)** | ***p*** |
| **Race/Ethnicity** |  |  |  |  |  |  |  |  |  |  |  |  |  |  |  |
| Mexican American |  | 1.02 (0.67, 1.54) | 0.932 | 0.91 (0.59, 1.41) | 0.668 | 0.79 (0.50, 1.26) | 0.319 |  |  | 1.02 (0.72, 1.44) | 0.903 | 0.84 (0.58, 1.23) | 0.377 | 0.79 (0.53, 1.17) | 0.241 |
| Other Hispanic | 0.226 | 1.54 (0.88, 2.68) | 0.129 | 1.37 (0.78, 2.41) | 0.274 | 1.54 (0.87, 2.75) | 0.138 |  | 0.987 | 1.03 (0.51, 2.08) | 0.937 | 0.90 (0.43, 1.88) | 0.770 | 0.82 (0.40, 1.69) | 0.591 |
| Non-Hispanic White | 0.990 | 1.01 (0.75, 1.37) | 0.924 | 0.95 (0.70, 1.29) | 0.727 | 0.93 (0.68, 1.27) | 0.644 |  | 0.582 | 0.91 (0.67, 1.23) | 0.536 | 0.83 (0.61, 1.13) | 0.234 | 0.85 (0.61, 1.20) | 0.354 |
| Non-Hispanic Black | 0.627 | 0.90 (0.68, 1.20) | 0.476 | 0.86 (0.65, 1.16) | 0.322 | 0.86 (0.63, 1.17) | 0.330 |  | 0.488 | 0.85 (0.61, 1.19) | 0.342 | 0.84 (0.59, 1.18) | 0.307 | 0.81 (0.56, 1.18) | 0.271 |
| Other Race-Including Multi-Racial | 0.678 | 0.87 (0.49, 1.55) | 0.636 | 0.80 (0.43, 1.48) | 0.468 | 0.78 (0.42, 1.44) | 0.419 |  | 0.720 | 1.16 (0.64, 2.12) | 0.615 | 1.01 (0.52, 1.96) | 0.968 | 0.90 (0.46, 1.74) | 0.745 |
| **Education (%)** |  |  |  |  |  |  |  |  |  |  |  |  |  |  |  |
| Below high school |  | 1.07 (0.68, 1.70) | 0.756 | 1.00 (0.63, 1.60) | 1.000 | 0.88 (0.54, 1.42) | 0.590 |  |  | 1.08 (0.66, 1.79) | 0.751 | 0.91 (0.54, 1.56) | 0.740 | 0.82 (0.48, 1.40) | 0.473 |
| High school Grad/GED or Equivalent | 0.765 | 1.18 (0.78, 1.79) | 0.417 | 1.06 (0.70, 1.60) | 0.775 | 1.08 (0.71, 1.64) | 0.725 |  | 0.710 | 1.22 (0.81, 1.85) | 0.342 | 1.05 (0.70, 1.58) | 0.814 | 1.17 (0.79, 1.73) | 0.437 |
| College or above | 0.626 | 0.94 (0.73, 1.22) | 0.636 | 0.91 (0.69, 1.19) | 0.477 | 0.88 (0.67, 1.16) | 0.362 |  | 0.404 | 0.86 (0.67, 1.10) | 0.225 | 0.80 (0.62, 1.05) | 0.110 | 0.80 (0.60, 1.06) | 0.118 |
| **PIR** |  |  |  |  |  |  |  |  |  |  |  |  |  |  |  |
| <1.0% |  | 1.15 (0.84, 1.60) | 0.380 | 1.12 (0.80, 1.59) | 0.504 | 1.14 (0.80, 1.63) | 0.468 |  |  | 1.04 (0.68, 1.60) | 0.859 | 0.99 (0.63, 1.56) | 0.976 | 0.93 (0.57, 1.51) | 0.768 |
| 1.0~2.0% | 0.761 | 1.08 (0.83, 1.41) | 0.560 | 0.99 (0.75, 1.30) | 0.915 | 0.92 (0.68, 1.24) | 0.570 |  | 0.609 | 1.18 (0.86, 1.62) | 0.308 | 1.04 (0.75, 1.44) | 0.806 | 1.04 (0.72, 1.49) | 0.846 |
| ≥2.0% | 0.279 | 0.93 (0.69, 1.24) | 0.604 | 0.87 (0.63, 1.18) | 0.363 | 0.87 (0.63, 1.19) | 0.372 |  | 0.339 | 0.82 (0.62, 1.08) | 0.157 | 0.73 (0.54, 1.00) | 0.050 | 0.75 (0.54, 1.04) | 0.088 |
| **BMI** |  |  |  |  |  |  |  |  |  |  |  |  |  |  |  |
| < 25 kg/m2 |  | 0.77 (0.46, 1.28) | 0.308 | 0.85 (0.51, 1.41) | 0.517 | 0.83 (0.51, 1.36) | 0.461 |  |  | 0.68 (0.41, 1.11) | 0.124 | 0.68 (0.42, 1.11) | 0.125 | 0.61 (0.36, 1.02) | 0.060 |
| 25~30kg/m2 | 0.309 | 1.07 (0.72, 1.59) | 0.736 | 0.98 (0.64, 1.52) | 0.944 | 0.90 (0.58, 1.39) | 0.627 |  | 0.458 | 0.88 (0.57, 1.34) | 0.546 | 0.80 (0.52, 1.25) | 0.327 | 0.69 (0.46, 1.03) | 0.071 |
| ≥30 kg/m2 | 0.381 | 1.01 (0.78, 1.31) | 0.941 | 0.96 (0.73, 1.28) | 0.800 | 0.95 (0.72, 1.26) | 0.714 |  | 0.164 | 0.99 (0.76, 1.29) | 0.941 | 0.92 (0.69, 1.25) | 0.604 | 0.96 (0.70, 1.30) | 0.766 |
| **WC** | 0.569 |  |  |  |  |  |  |  | 0.825 |  |  |  |  |  |  |
| Normal waist circumference |  | 1.13 (0.71, 1.79) | 0.606 | 1.17 (0.73, 1.88) | 0.499 | 1.13 (0.70, 1.81) | 0.620 |  |  | 0.87 (0.53, 1.41) | 0.563 | 0.88 (0.54, 1.44) | 0.611 | 0.74 (0.44, 1.24) | 0.251 |
| Abdominal obesity |  | 0.96 (0.77, 1.21) | 0.747 | 0.90 (0.71, 1.15) | 0.405 | 0.88 (0.69, 1.12) | 0.306 |  |  | 0.92 (0.74, 1.16) | 0.492 | 0.85 (0.66, 1.10) | 0.205 | 0.87 (0.66, 1.14) | 0.318 |
| **Smoking** | 0.253 |  |  |  |  |  |  |  | 0.105 |  |  |  |  |  |  |
| Never smoking |  | 1.12 (0.85, 1.47) | 0.424 | 1.06 (0.79, 1.43) | 0.691 | 0.99 (0.74, 1.33) | 0.966 |  |  | 0.76 (0.55, 1.04) | 0.083 | 0.73 (0.53, 1.01) | 0.058 | 0.66 (0.47, 0.92) | 0.016 |
| Smoker |  | 0.91 (0.71, 1.16) | 0.435 | 0.85 (0.66, 1.10) | 0.223 | 0.84 (0.65, 1.09) | 0.195 |  |  | 1.10 (0.83, 1.45) | 0.505 | 1.00 (0.75, 1.34) | 0.998 | 1.00 (0.72, 1.38) | 0.998 |
| **Hypertension** | 0.604 |  |  |  |  |  |  |  | 0.432 |  |  |  |  |  |  |
| No |  | 0.91 (0.66, 1.27) | 0.583 | 0.80 (0.57, 1.12) | 0.186 | 0.80 (0.57, 1.12) | 0.195 |  |  | 1.04 (0.73, 1.47) | 0.845 | 0.82 (0.57, 1.19) | 0.291 | 0.81 (0.56, 1.18) | 0.273 |
| Yes |  | 1.01 (0.81, 1.27) | 0.918 | 1.01 (0.79, 1.28) | 0.955 | 0.98 (0.77, 1.25) | 0.859 |  |  | 0.87 (0.68, 1.11) | 0.258 | 0.89 (0.68, 1.15) | 0.357 | 0.81 (0.62, 1.06) | 0.130 |
